# Melanoma incidence in Norway, 1980–2024: a cohort study

**DOI:** 10.1016/j.lanepe.2026.101882

**Published:** 2026-09-30

**Authors:** Saha Samimi, Trude E. Robsahm, Reza Ghiasvand

**Affiliations:** a Oslo Centre for Biostatistics and Epidemiology, Oslo University Hospital, Oslo, Norway; b Faculty of Medicine, Institute for Medical Information Processing, Biometry and Epidemiology (IBE), LMU, Munich, Germany; c Department of Research, Cancer Registry of Norway, Norwegian Institute of Public Health, Oslo, Norway

**Keywords:** Melanoma, Incidence, Age-period-cohort, Norway, Joinpoint, Trend analysis

## Abstract

**Background:**

Norway has one of the world's highest melanoma incidence rates. Despite preventive efforts, their long-term impact remains unclear. We examined sex- and age-specific melanoma trends in Norway.

**Methods:**

We used nationwide Cancer Registry data on first primary melanoma in Norway during 1980–2024. Incidence rates were age-standardised to the European Standard Population 2013. Joinpoint regression estimated average annual percentage changes (AAPCs) with 95% confidence intervals (CIs), by sex, age, region, anatomical site, and subtype. Age-period-cohort analyses assessed temporal patterns.

**Findings:**

Among 60,722 individuals with melanoma, 29,775 (49.0%) were male. Overall, incidence increased by 2·4–2·7% yearly in 1980–2024 in both sexes. Trends diverged, with stable or declining incidence among younger individuals and continued increases exceeding 5% [5·2, 6·6] in the oldest groups. Birth-cohort analyses identified a strong birth-cohort effect, with progressively increasing incidence across successive older birth cohorts, whereas younger cohorts showed stable or declining incidence. Northern Norway had the largest regional increases (AAPC 4·6 [3·8, 5·3] in males and 3·1 [2·5, 3·8] in females). Incidence increased particularly on the head and neck in men and the trunk and upper limbs in women. Superficial spreading melanoma showed the strongest increase among subtypes (4·1 [3·7, 4·6] and 3·1 [2·8, 3·5], respectively).

**Interpretation:**

Melanoma incidence in Norway continues to rise mainly among older adults, while trends in younger generations are stabilising or declining. Cohort patterns and regional differences are consistent with potential changes in UVR-related behaviours. These findings support the need for targeted prevention strategies for groups with persistently increasing incidence.

**Funding:**

None.

**Research in context**

**Evidence before this study**

Previous studies from Australia, New Zealand, the USA, the UK, and Scandinavia have reported declining or stabilising melanoma incidence among younger individuals, while incidence has increased among older adults. In Norway, a nationwide study reported a more than seven-fold increase between 1954 and 2008, with the steepest increases among individuals older than 50 years. We identified relevant studies by searching MEDLINE/PubMed from database inception to July 2026 using combinations of “cutaneous melanoma”, “malignant melanoma”, “melanoma incidence”, “temporal trends”, “age-period-cohort”, “birth cohort”, and “epidemiology”. We also reviewed reports and databases from the International Agency for Research on Cancer, NORDCAN, and Nordic national melanoma registries. Norway has one of the highest melanoma incidence rates worldwide and represents a distinct epidemiological setting, characterised by a predominantly fair-skinned population, intermittent recreational ultraviolet radiation (UVR) exposure patterns, and no nationwide, sustained government-funded melanoma prevention programme. However, no previous nationwide study has comprehensively examined long-term incidence trends by age, sex, region, anatomical site, and melanoma subtype together with birth-cohort patterns.

**Added value of this study**

This nationwide registry-based study provides a comprehensive analysis of first primary melanoma incidence in Norway over 45 years, including 60,722 individuals diagnosed between 1980 and 2024. Using Joinpoint and age-period-cohort analyses, we examined temporal patterns. We found that melanoma incidence increased substantially over time, driven mainly by older age groups and particularly by males, while incidence among individuals younger than 40 years remained stable or declined in recent decades. We identified marked birth-cohort effects, with progressively increasing incidence across older generations, but stable or declining incidence in younger cohorts. Additionally, the steepest increases were observed in northern Norway and for superficial spreading melanoma. Sex-specific differences by anatomical site were also observed.

**Implications of all the available evidence**

Our findings suggest that generational patterns in melanoma incidence in Norway are changing, with stabilising trends in younger age groups but continued increases among older cohorts, particularly among males. The regional and anatomical-site differences, including the steep increase in northern regions despite lower ambient UVR, are consistent with changing UVR-related behaviours over time, including sunbathing, indoor tanning, and travel to high-UVR destinations. Differences in cohort-specific patterns may reflect cumulative lifetime exposure. These findings support targeted prevention strategies aimed at reducing UVR overexposure among high-risk groups and regions, alongside continued risk-adjusted surveillance of cohort-specific trends.

## Introduction

Cutaneous melanoma (hereafter melanoma) is one of the most common cancers in white populations and accounts for most skin cancer deaths, with incidence rising substantially worldwide.^1^^,^^2^ According to the World Health Organization's International Agency for Research on Cancer, there were 331,722 new melanoma diagnoses and 58,667 deaths worldwide in 2022.^3^ The highest rates are observed in Oceania, particularly Australia, North America and Western and Northern Europe.^3^, ^4^, ^5^ In Europe, there were 146,321 new cases, and 26,180 deaths due to melanoma in 2022.^3^ Overall, the continent has an incidence of 10·4 per 100,000, with Norway reporting the second highest rate at 30·6 per 100,000.^4^^,^^6^ In Norway, melanoma incidence has increased more than sevenfold over the past six decades, now ranking as the third highest worldwide.^3^^,^^7^

Ultraviolet radiation (UVR) is the main cause of melanoma, inducing DNA damage and immunological alterations.^1^ Due to its proven cancer-causing effects, UVR is classified as a Group 1 carcinogen by the International Agency for Research on Cancer.^8^ The increase in melanoma incidence in recent decades has largely been attributed to increasing sunlight exposure.^2^ Melanoma risk increases with the number of sunburns over the life course,^9^, ^10^, ^11^ high-intensity intermittent sun exposure,^1^^,^^9^ and use of indoor tanning devices, particularly among younger adults and women.^10^ While UVR remains the key target for preventive interventions, other established risk factors for melanoma incidence include red hair, freckles, or light/fair skin—often corresponding to skin phototypes I or II on the Fitzpatrick scale—proneness to sunburn, genetic background, and a high number of nevi.^1^^,^^10^, ^11^, ^12^

Worldwide, several interventions have been implemented to counteract the rising melanoma incidence, with a primary focus on reducing UVR radiation exposure and promoting early detection.^1^^,^^13^^,^^14^ Australia has established the most comprehensive prevention programme, initiated in the 1980s and comprising coordinated, multisectoral measures, including public education campaigns, school-based initiatives, and regulatory actions. Recent data from Australia, the UK, the USA, and Sweden have shown decreasing incidence in younger age groups,^15^, ^16^, ^17^, ^18^, ^19^ although the contribution of prevention efforts is difficult to quantify. In Norway, the main skin cancer prevention efforts have been conducted by the Norwegian Cancer Society and the Norwegian Radiation and Nuclear Safety Authority through public campaigns and guidance on sun protection since the 1990s. However, evidence suggests that these initiatives have not necessarily been translated into sustained sun-protective behaviours. In a large cohort study of nearly 150,000 Norwegian women, sunscreen use rose between 1997 and 2007, yet the proportion reporting at least one sunburn per year increased from 39% to 46% over the same period.^20^ It remains unclear whether melanoma incidence trends in Norway show patterns compatible with sustained changes in UVR-related behaviour, particularly in younger age groups.

Understanding long-term changes in melanoma incidence in the context of decades of prevention efforts is important to guide future policy. Thus, this study aims to examine long-term sex- and age-specific trends in incidence of first primary melanoma between 1980 and 2024 in Norway, by region, anatomical site, and melanoma subtype using data from the Cancer Registry of Norway.

## Methods

### Study data

In this national registry-based study, we used data from the Cancer Registry of Norway on diagnoses of first primary invasive melanoma, according to the International Classification of Diseases, 10th edition (ICD-10 code C43)^21^ between the years 1980 and 2024 in Norway. Aggregated data are available from the Norwegian Cancer Registry upon request, which is responsible for registering all cancer diagnoses except basal cell carcinoma. Mandatory reporting of new cases of cancer in Norway results in a close-to-complete rate of 98·8%.^22^ We obtained group-level data on age at diagnosis (categorised as: 10–29 years, 30–39 years, 40–49 years, 50–59 years, 60–69 years, 70–79 years, 80+ years), sex (male, female), pre-defined health region, which also corresponds to residential ambient UVR intensity (south-eastern [highest], western [medium], central [medium-low], and northern [lowest] Norway), anatomic site according to the ICD-10 codes (head and neck [C43.0–43.4], trunk [C43.5], upper limbs [C43.6], lower limbs [C43.7], and skin unspecified [C43.8, C43.9]),^21^ and melanoma subtype according to ICD-O-3 codes (superficial spreading melanoma (8743/3), nodular melanoma (8721/3), unspecified (8720/3), and other melanoma (other melanoma-specific codes)). Due to changes in coding practices, analyses by anatomic site (1994–2024) and melanoma subtype (1984–2024) were conducted over periods that differ from the overall interval (1980–2024). We additionally obtained aggregated data from Statistics Norway on the proportion of immigrants and Norwegian-born individuals with immigrant parents, by region and country/region of origin, for 1980–2024, to provide context for interpreting regional incidence trends.

### Statistical analysis

The study involved descriptive analyses of frequencies (N and %) and Joinpoint analysis was used to calculate segment-specific Annual Percent Change (APC) and Average Annual Percent Change (AAPC), which summarise the segment-specific APCs over the entire analysis period, with 95% confidence intervals (CI) based on log-transformed age-standardised incidence rates. Rates were standardised to the European Standard Population 2013 and calculated as the number of cases in each group divided by population at risk (per 100,000 person-years). Joinpoint analyses were used to estimate long-term trends in sex- and age-specific incidence rates by region, anatomical site, and melanoma subtype. Additionally, an age-period-cohort analysis was conducted to examine temporal trends across different birth cohorts. For visualisation, cohort-specific incidence rates were smoothed separately by sex and age group using LOESS (locally weighted regression) with a span of 0·5. The time period was segmented into two periods (1980–2005 and 2006–2024) to ensure comparability with the results of previous studies conducted in countries with a similar risk profile. The Joinpoint software version 5.4.0 (April 2025) computed a piecewise linear regression and best-fitting breakpoints, and was provided by the US National Cancer Institute.^23^ All other statistical analyses were performed in R version 4.5.1 (June 2025) with p < 0·05 considered statistically significant.^24^

### Ethical approval

The use of de-identified aggregated data and the analyses presented in this manuscript fall under the purview of the Cancer Registry of Norway's mandate and approval to provide delivery of statistics.

### Role of the funding source

There was no funding source for this study.

## Results

A total of 60,722 individuals, including 29,775 (49·0%) males and 30,947 (51·0%) females with a first primary melanoma were registered in the Cancer Registry of Norway during the 45-year study period (1980–2024) (Table 1). Among melanoma cases, the highest proportion of new melanoma cases was observed in individuals aged ≥60 years, accounting for approximately 62·3% (n = 18,528) of all males and 53·5% (n = 16,527) of all female melanoma cases, whereas those aged <30 years represented 2·3% (n = 682) and 4·8% (n = 1483), respectively. Among males, melanoma occurred most commonly on the trunk (49·4%, 12,495/25,278), the upper limbs (17·5%, 4436/25,278), and head and neck (15·0%, 3804/25,278), whereas among females, lower limbs (33·3%, 8456/25,373), trunk (29·6%, 7514/25,373), and upper limbs (21·1%, 5357/25,373) were most affected. Regarding histological subtype, most cases (15,932/28,820 (55·3%) in males and 17,690/29,675 (59·6%) in females) were classified as superficial spreading melanoma, followed by nodular melanoma (5510/28,820 (19·1%) and 4650/29,675 (15·7%), respectively) and other subtypes (7378/28,820 (25·6%) and 7335/29,675 (24·7%), respectively).

**Table 1:** Average annual percent change (AAPC) with 95% confidence intervals (CI) in sex-specific melanoma incidence in Norway, by patient characteristics, during 1980–2024, and tumour characteristics, subtype, 1993–2024, and anatomic site, 1994–2024.

Variable	Males		Females	
	N (%)	AAPC (95% CI)	N (%)	AAPC (95% CI)
**Overall**	**29,775 (49.0%)**	2·7 (2·4, 3·0)^a^	**30,947 (51·0%)**	2·4 (2·2, 2·7)^a^
Age group (years)	**29,764 (49·0%)**^b^	2·7 (2·4, 3·0)^a^	**30,938 (51·0%)**^b^	2·4 (2·2, 2·7)^a^
10–29	682 (2·3%)	−0·7 (−2·5, 1·1)	1483 (4·8%)	−0·7 (−1·7, 0·2)
30–39	1711 (5·8%)	−0·2 (−0·7, 0·4)	2824 (9·1%)	0·0 (−0·3, 0·3)
40–49	3435 (11·5%)	0·9 (0·4, 1·3)^a^	4522 (14·6%)	1·1 (0·7, 1·6)^a^
50–59	5408 (18·2%)	2·6 (2·1, 3·0)^a^	5582 (18·0%)	2·6 (2·1, 3·3)^a^
60–69	7042 (23·7%)	3·5 (2·9, 4·1)^a^	6093 (19·7%)	3·0 (2·7, 3·3)^a^
70–79	7202 (24·2%)	4·8 (4·4, 5·1)^a^	5918 (19·1%)	3·9 (3·6, 4·1)^a^
80+	4284 (14·4%)	5·9 (5·2, 6·6)^a^	4516 (14·6%)	4·1 (3·6, 4·7)^a^
NA	11 (0·0%)		9 (0·0%)	
Region	**29,775 (49·0%)**^b^		**30,947 (51·0%)**^b^	
South-East	18,185 (61·1%)	2·4 (2·0, 2·8)^a^	18,656 (60·3%)	2·2 (1·9, 2·5)^a^
West	6113 (20·5%)	3·0 (2·7, 3·3)^a^	6523 (21·1%)	2·2 (1·9, 2·5)^a^
Central	3730 (12·5%)	2·8 (2·5, 3·2)^a^	4011 (13·0%)	2·6 (2·1, 3·0)^a^
North	1747 (5·9%)	4·6 (3·8, 5·3)^a^	1757 (5·7%)	3·1 (2·5, 3·8)^a^
Anatomic site	**25,278 (49·9%)**^b^		**25,373 (50·1%)**^b^	
Head and neck	3804 (15·0%)	3·1 (2·5, 3·6)^a^	3040 (12·0%)	2·1 (1·6, 2·7)^a^
Trunk	12,495 (49·4%)	2·7 (2·3, 3·1)^a^	7514 (29·6%)	3·3 (2·8, 3·7)^a^
Upper limbs	4436 (17·5%)	2·7 (2·0, 3·8)^a^	5357 (21·1%)	3·4 (2·9, 3·8)^a^
Lower limbs	3096 (12·2%)	2·1 (1·2, 3·0)^a^	8456 (33·3%)	1·6 (1·0, 2·4)^a^
Skin unspecified	1447 (5·7%)	1·7 (0·8, 2·7)^a^	1006 (4·0%)	1·0 (−0·4, 2·4)
NA/suppressed^c^	4497 (17·8%)		5574 (22·0%)	
Melanoma type	**28,820 (49·4%)**^b^		**29,675 (50·6%)**^b^	
Superficial spreading melanoma	15,932 (55·3%)	4·1 (3·7, 4·6)^a^	17,690 (59·6%)	3·1 (2·8, 3·5)^a^
Nodular melanoma	5510 (19·1%)	1·5 (0·9, 2·2)^a^	4650 (15·7%)	0·5 (−0·2, 1·6)
Other types	7378 (25·6%)	2·7 (2·2, 3·1)^a^	7335 (24·7%)	2·1 (1·6, 2·4)^a^
NA/suppressed^c^	955 (3·3%)		1272 (4·3%)	

Bold numbers (%) represent the total number and percentage for each variable

a p-value <0·05.

b Slight discrepancies in the total number are attributable to missing values in some variables.

c Annual case counts were suppressed when numbers were sufficiently small to pose a potential risk of individual identification.

### Trends in incidence by sex and age

Melanoma incidence increased from 12·4 per 100,000 in 1980 to 35·7 in 2024 among males, and from 15 to 33·6 among females (Figure S1). Over the same period, incidence increased by 2·7% per year [95% CI: 2·4, 3·0] in males and by 2·4% [2·2, 2·7] in females (Table 1, Figure S1). Trends differed markedly by age. Incidence was low and stable, or tended to decline, in individuals younger than 40 years in both sexes, with higher rates among females than males in these age groups (Fig. 1, Table S1, Figure S2). From age 40 years onwards, incidence increased, and from age 50 years the increases were statistically significant in both sexes, with the steepest annual increases observed among individuals aged 70 years or older (Table 1, Fig. 1, Table S2). After age 70 years, incidence rates were consistently higher in males than in females (Fig. 1, Figure S2).

**Fig. 1:**
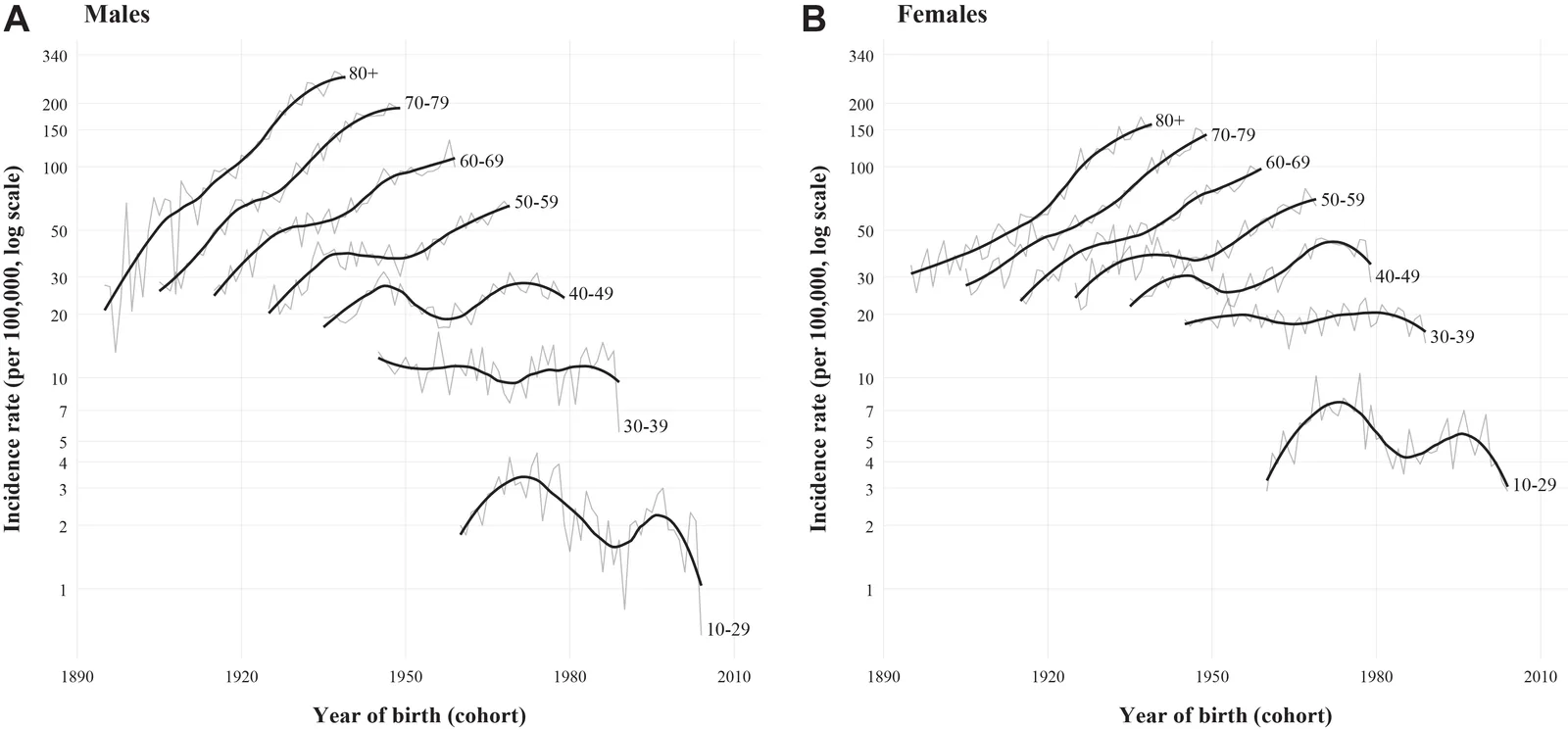
Birth-cohort analysis of melanoma incidence in Norway, by age group and sex, during 1980–2024. (A) Males; (B) Females.

The age-related pattern persisted when the study period was analysed separately for 1980–2005 and 2006–2024 (Fig. 2, Table S2). During 2006–2024, AAPCs were negative or close to zero in individuals younger than 40 years, remained stable among those aged 40–49 years, and increased progressively from age 50 years onwards, exceeding 5% per year in the oldest age groups (5·33 [3·79, 7·06] in males and 5·11 [3·80, 6·38] in females).

**Fig. 2:**
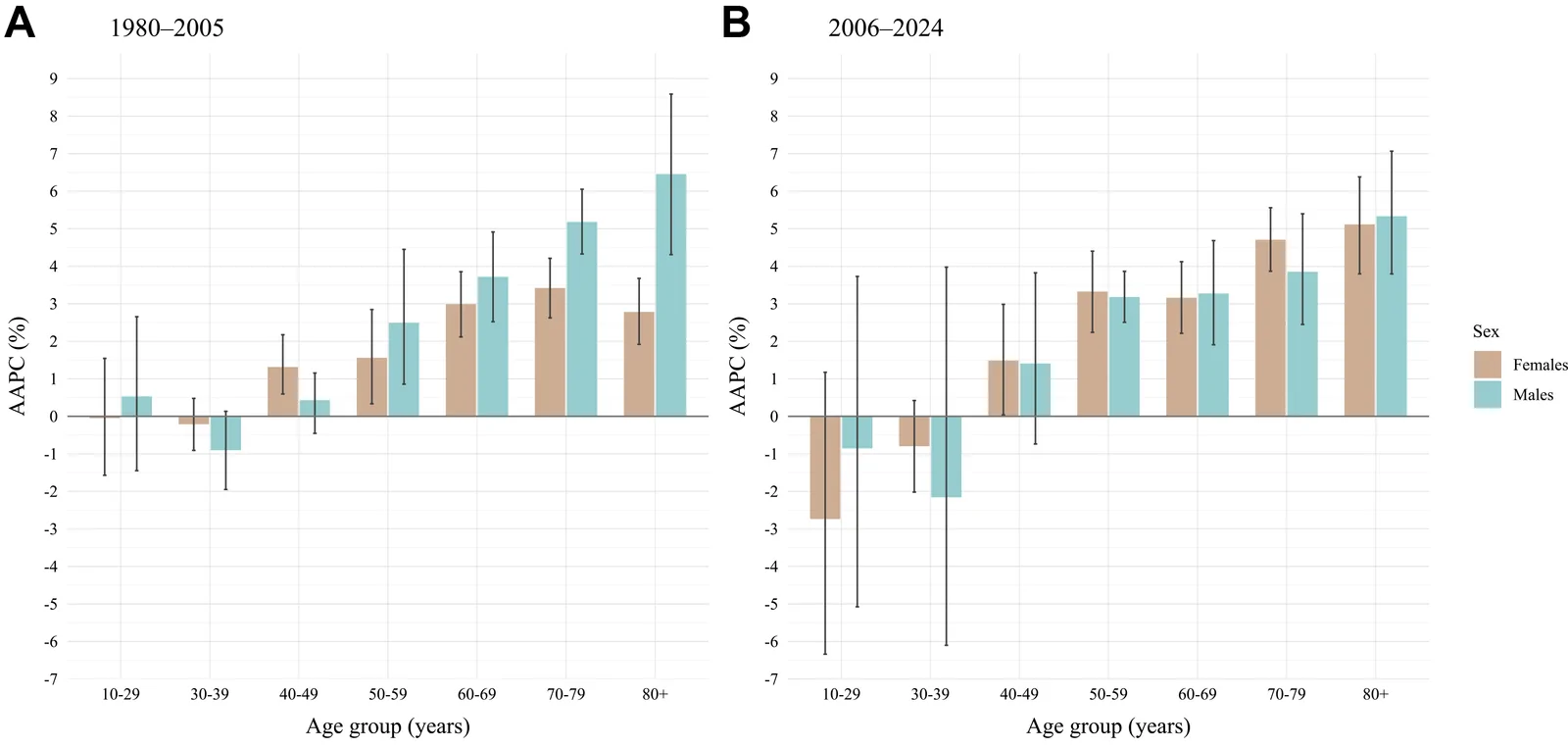
Average annual percent change (AAPC) with 95% confidence intervals (CI) in melanoma incidence in Norway, by sex and age group, for the period 1980–2005 (A) and 2006–2024 (B).

The birth-cohort analysis revealed a clear cohort effect, with progressively higher melanoma incidence observed in successively more recent birth cohorts among individuals older than 40 years, particularly in those aged ≥60 years, in both sexes. In contrast, younger birth cohorts exhibited comparatively lower and more stable incidence patterns in age groups younger than 40 years, with evidence of plateauing or modest declines (Fig. 1). Period analyses further demonstrated a significant temporal increase in melanoma incidence from age group 40–49 onward across successive calendar periods (Fig. 3). In age groups 50 years or older, recent period-specific APCs were generally higher than the AAPCs for the full study period (1980–2024; Table 1), indicating accelerated increases in the most recent decades (2006–2024).

**Fig. 3:**
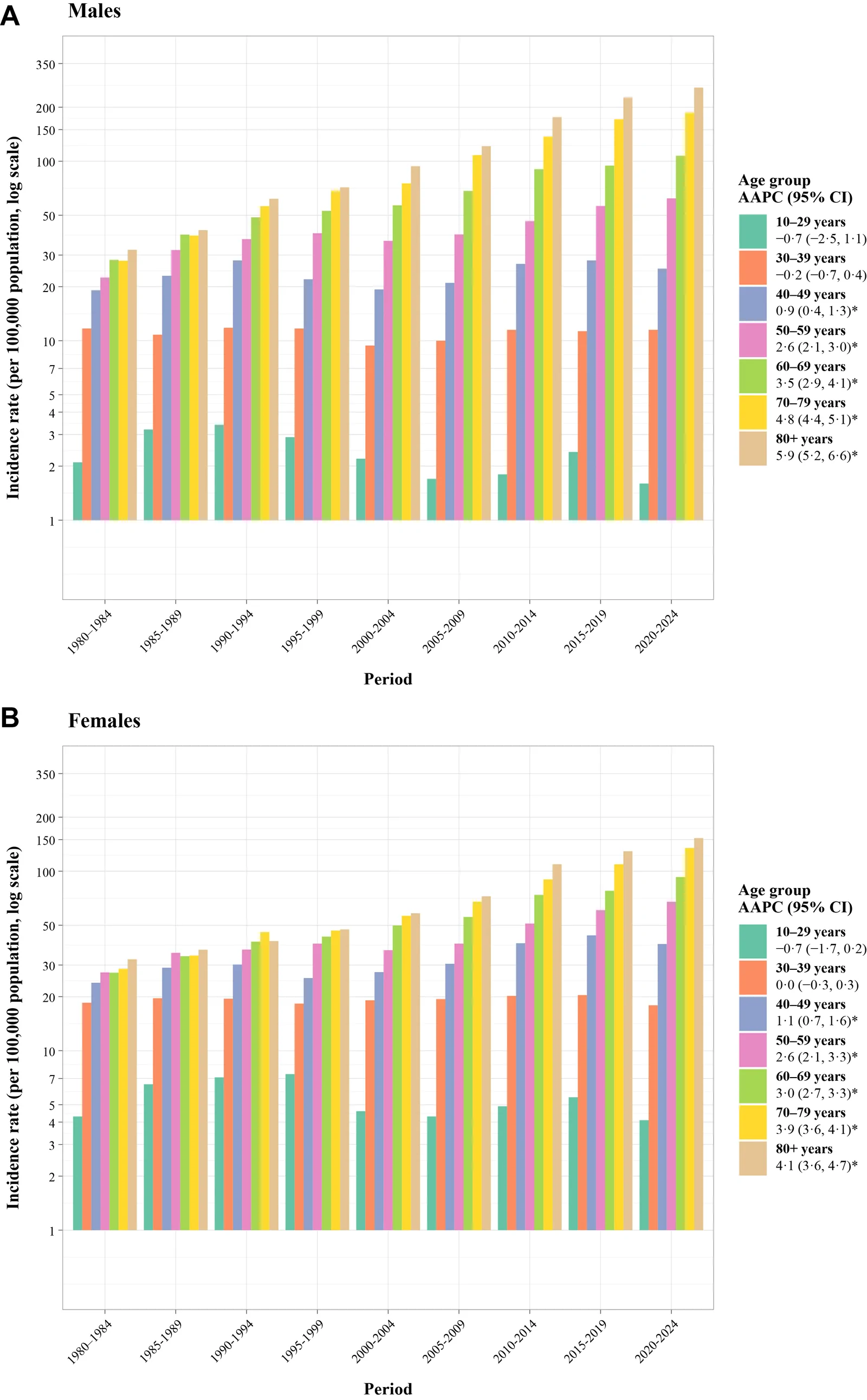
Age-specific incidence rates of melanoma in Norway, by sex during 1980–2024. (A) Males; (B) Females.

### Trends in incidence by region

During the entire period (1980–2024), the northern region of Norway experienced the largest increase, with an AAPC of 4·6 [3·8, 5·3] in males and 3·1 [2·5, 3·8] in females, while the increase was smaller in the central (2·8 [2·5, 3·2] and 2·6 [2·1, 3·0], respectively), the south-eastern (2·4 [2·0, 2·8] and 2·2 [1·9, 2·5], respectively), and the western region (3·0 [2·7, 3·3] and 2·2 [1·9, 2·5], respectively) (Table 1). Fig. 4 shows period-specific changes by region, with the corresponding AAPCs reported in Table S2. Between 1980 and 2005, the largest incidence increase was observed in northern Norway among males (AAPC 4·6 [1·5, 7·8]) and in western Norway among females (AAPC 2·2 [1·3, 3·1]), corresponding to an incidence increase from 3·2 and 9·9 per 100,000 in 1980 to 23·9 and 24·5 per 100,000 in 2024, respectively (Supplementary Material, Table S3). During the second period (2006–2024), the overall pattern persisted in males, while among females, the steepest rise was seen in northern Norway (AAPC 5·3 [3·5, 7·1]). Although the northern region showed the steepest relative increase, the South-East continued to account for the largest absolute burden of melanoma because of its substantially higher population. Across all regions for both sexes, incidence increased more rapidly during 2006–2024 than during 1980–2005. The proportion of immigrants and Norwegian-born individuals with immigrant parents increased across all regions during the study period, with individuals of European origin remaining the largest origin group (Supplementary Material, Table S5).

**Fig. 4:**
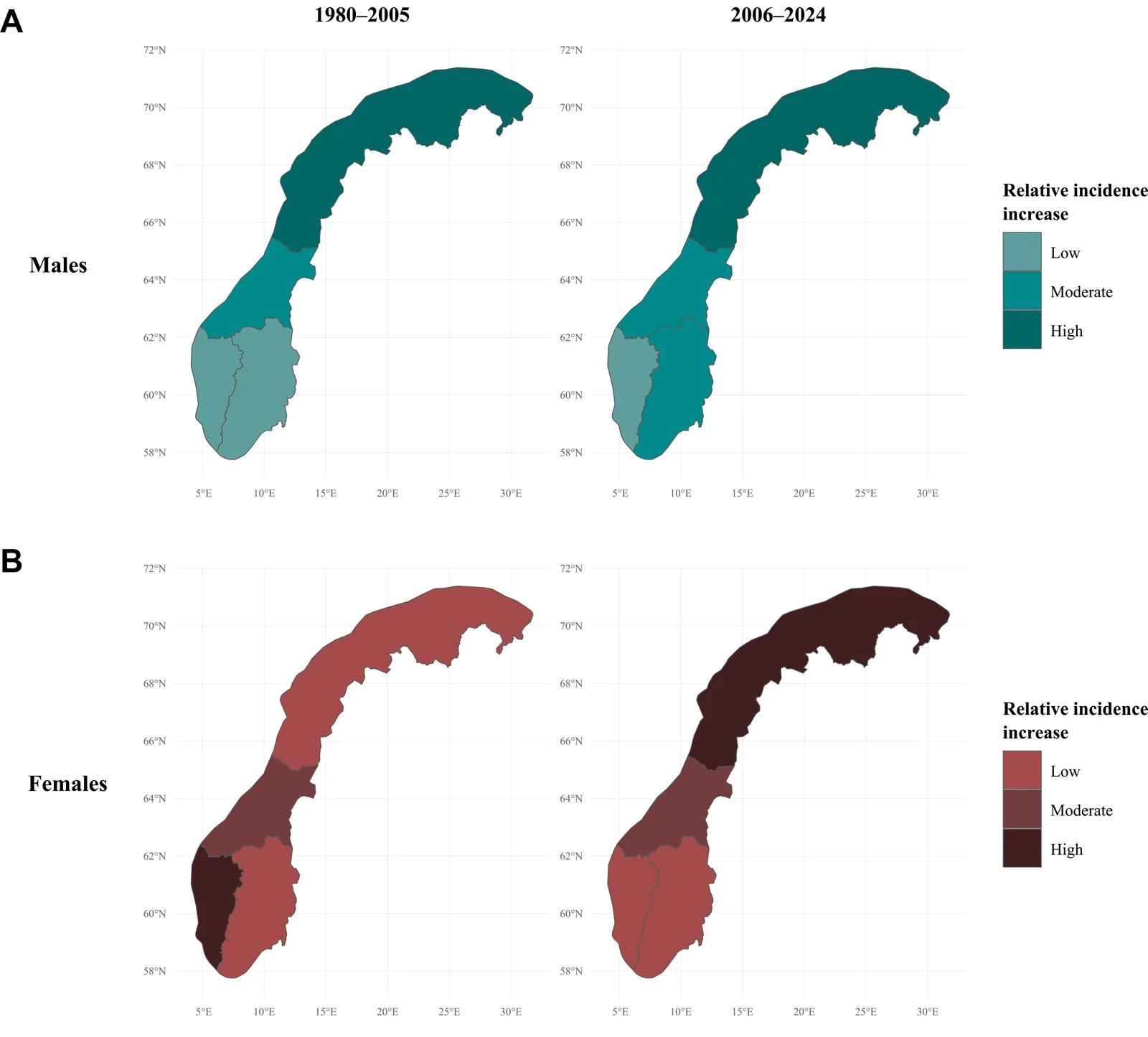
Relative incidence increase in melanoma incidence in Norway during 1980–2005 and 2006–2024, by region.

### Trends in incidence by anatomic site and subtype

Between 1994 and 2024, incidence increased substantially on the trunk (AAPC 2·7 [2·3, 3·1] in males and 3·3 [2·8, 3·7] in females) and on the upper limbs (2·7 [2·0, 3·8] and 3·4 [2·9, 3·8], respectively) (Table 1). Among males, a large increase was also observed for head and neck melanomas (AAPC 3·1 [2·5, 3·6]). In the analysis by histological subtype, the incidence of superficial spreading melanoma increased markedly between 1984 and 2024 in both males (AAPC of 4·1 [3·7, 4·6]) and females (AAPC 3·1 [2·8, 3·5]), whereas nodular melanoma increased moderately in males (AAPC 1·5 [0·9, 2·2]). When stratified by age, superficial spreading melanoma increased modestly among individuals younger than 50 years (AAPC 0·7 [0·2, 1·2]) and more markedly among those aged 50–69 years (3·8 [3·4, 4·5]) and ≥70 years (6·0 [5·5, 6·7]). Nodular melanoma decreased among individuals younger than 50 years (−2·2 [−3·1, −1·5]) but increased among those aged ≥70 years (2·9 [2·3, 3·4]). Other subtypes also showed increasing incidence among individuals aged 50–69 years (1·7 [1·2, 2·2]) and ≥70 years (3·7 [3·0, 4·3]) (Supplementary Material, Table S4).

## Discussion

This nationwide study examined long-term sex- and age-specific trends in first primary melanoma incidence in Norway by region, anatomical site, and melanoma subtype. Over the 45-year study period, melanoma incidence increased by more than 2% per year on average in both sexes, with stable or declining trends among individuals younger than 40 years and increasing incidence across older age groups. Notably, age-period-cohort analyses indicated progressively increasing incidence across successive birth cohorts among individuals aged 40 years and older, particularly those aged 60 years and older, while incidence in individuals younger than 40 years tended to decline. The analyses also identified marked differences by region, anatomical site, and melanoma subtype.

The substantial increase in melanoma incidence among older age groups observed in this study aligns with the long latency of melanoma development and the cumulative effect of UVR exposure over the life course.^1^^,^^9^, ^10^, ^11^ The steeper rise among older males is consistent with behavioural factors, including differences in sun-protective practices, resulting in greater cumulative or chronic UVR exposure.^25^^,^^26^ The pronounced birth-cohort pattern is a central finding of this study, with incidence increasing across successive birth cohorts in older age groups, particularly from age 60 years onwards. These findings extend previous reports of age-specific trends by demonstrating that the divergence between younger and older adults is accompanied by a pronounced birth-cohort effect, suggesting that melanoma risk has changed systematically across generations. Age-specific trends from the USA, the UK, Scandinavia, and multicountry European studies have shown similar patterns since 2000 in both sexes.^15^, ^16^, ^17^, ^18^, ^19^ By contrast, Australia has not shown the same degree of increase with advancing age, and overall trends have been more modest.^16^

Our findings also indicate a decreasing trend in individuals younger than 40 years in the past two decades in line with studies from Europe and the US, while this decreasing trend has been more pronounced in Australia.^15^, ^16^, ^17^^,^^19^ These differences might reflect variation in the timing, intensity, and population reach of national prevention strategies. Australia's comprehensive, long-standing programme, including mass-media campaigns such as the SunSmart programme, sunbed regulation, and initiatives targeting both primary prevention and early detection, may have contributed to improved sun-protective behaviours and the stabilisation or decline in melanoma incidence among younger age groups.^13^^,^^14^ However, observed increasing time spent on screen-based activities^27^ may have displaced time spent on outdoor play and thus contributed to this, which also might have influenced melanoma rates in Norway where adolescents now spend 6 h per day on screen-based activities.^28^

Melanoma overdiagnosis has been suggested as one possible contributor to the increasing incidence observed in fair-skinned populations. A recent nationwide analysis of Norwegian data examined incidence trends by Breslow thickness (1983–2019) and found increasing incidence across virtually all thickness categories, including both thin (T1) and thicker (T3–T4) melanomas.^29^ These findings suggest that the rising melanoma incidence in Norway cannot be explained solely by increased detection of thin melanomas. Taken together with the age-period-cohort findings from the present study, including progressively increasing incidence across successive older birth cohorts and continued increases among older adults, this suggests that the observed trends are unlikely to be explained by diagnostic intensity alone.

In Norway, the observed differences by age, sex, and region identified in this study provide a basis for tailoring future prevention strategies to population groups at highest risk. The persistent rise in incidence despite decades of public awareness efforts by the Norwegian Cancer Society and the Radiation and Nuclear Safety Authority may suggest that behavioural change has been uneven. This is consistent with evidence from Norwegian women showing increased sunscreen use without a corresponding decline in sunburns,^20^ indicating a need for public health efforts beyond awareness alone to promote effective sun-protective behaviour. Given the central role of UVR exposure in melanoma development, particularly in early life,^10^, ^11^, ^12^ prevention policies should continue to address modifiable exposures, especially indoor tanning. Although the 2017 ban on sunbed use for individuals younger than 18 years in Norway represented an important regulatory measure,^30^ early evaluations suggest limited behavioural impact.^31^

Shifts in regional patterns provide additional insight into changing melanoma epidemiology in Norway. Although the south-eastern region with the highest ambient UVR accounts for the largest number of cases, the steepest increase was observed in northern Norway, particularly in recent decades. The steep increase in northern Norway despite lower ambient UVR suggests that changes in factors beyond local ambient UVR may have contributed to the observed trend. Agredano et al. reported an association between increased air passenger mileage and melanoma incidence in Norway,^32^ suggesting that access to international travel may be one contributing factor through greater intermittent sun exposure. This may partly reflect the increase in travel to sunny destinations that started in the 1970s.^32^

Immigration of low-risk individuals might also influence the incidence trends. Immigrants from Asia and Africa have constituted a relatively stable proportion of the immigrant population in the past decades, and individuals of European origin remain the largest immigrant group in Norway, thus the overall population susceptibility has remained largely unchanged.^33^

Behavioural differences may also explain the observed sex-specific patterns by anatomic site. Among males, the largest increase was observed for head and neck melanoma, a pattern generally considered consistent with more chronic sun exposure, including outdoor occupational activities.^25^^,^^26^ Among females, the largest increases were seen on the upper limbs and trunk, sites more often linked to intermittent exposure and potentially consistent with behaviours such as indoor tanning.^25^^,^^26^ However, the present location-specific results should be interpreted as descriptive incidence patterns only, and do not allow conclusions about underlying behavioural, diagnostic, or demographic mechanisms.

### Strengths and limitations

To our knowledge, this study is the first to use nationwide registry data to comprehensively assess long-term melanoma incidence trends in Norway across sex, age, birth cohorts, region, anatomic site, and subtype over more than four decades. The use of population-based data with high completeness provides a strong basis for reliable analysis of temporal patterns at the national level, covering more than 60,000 individuals diagnosed with a first primary melanoma. However, this observational study cannot establish causal associations. Although a recent nationwide Norwegian study demonstrated increasing incidence across both thin and thick melanomas, suggesting that the rising incidence cannot be explained solely by increased detection of thin lesions, the relative contributions of changes in disease occurrence, prevention efforts, public awareness and UVR-related behaviour, diagnostic intensity, and pathology practices to the temporal trends cannot be disentangled in the present study. Furthermore, incidence estimates in the youngest age groups are based on comparatively few cases and are therefore more prone to fluctuation. Additionally, more detailed stratified analyses were not presented, as the resulting small cell counts could pose a risk of individual identification. Due to its design, this study could not directly assess individual-level risk factors such as sun exposure, indoor tanning, travel to high-UVR destinations, sunscreen use, or screening behaviour. Finally, quantitative measurements of the skin cancer prevention efforts undertaken by the Norwegian Cancer Society and the Radiation and Nuclear Safety Authority are not available.

### Conclusion

In conclusion, melanoma incidence in Norway increased substantially between 1980 and 2024, with the strongest increase occurring in older age groups, particularly among males, and with notable differences by region, anatomic site, and subtype. The observed birth-cohort patterns are compatible with a potential generational change in UVR-related behaviours, consistent with stabilising incidence in younger adults despite continued increases among older age groups. Continued and targeted public health action is needed to reduce harmful UVR overexposure and the future burden of melanoma, particularly among older adults and in regions with steeply increasing incidence.

## Contributors

SS: data curation, formal analysis, methodology, writing original draft, review, and editing.

TER: conceptualisation, methodology, review, and editing, supervision.

RG: conceptualisation, methodology, review, and editing, supervision.

SS, TER and RG directly accessed and verified the data. All authors had full access to all the data in the study and had final responsibility for the decision to submit for publication.

## Data sharing statement

The Cancer Registry of Norway is responsible for registering all cancer diagnoses in Norway, except basal cell carcinoma, and provides aggregated data upon request.

## Declaration of interests

The authors declare no competing interests.
