# Supplementary material for: Melanoma incidence in Norway, 1980–2024: a cohort study
Source: Lancet Reg Health Eur. 2026 Sep 30;71:101882. doi: 10.1016/j.lanepe.2026.101882 (PMC13654556; doi:10.1016/j.lanepe.2026.101882)

Supplementary Materials

Contents

Tables 2

Table S1: Annual percent change (with 95% CI) in melanoma incidence in Norway, by age group, for males and females, during 1980-2024. Year intervals correspond to segments identified by joinpoint analysis. 2

Table S2: Average annual percent change (with 95% CI) in melanoma incidence in Norway, by sex, age group and region, during 1980-2005 and 2006-2024. 3

Table S3: Region-specific incidence rates of melanoma per 100,000, by sex, in 1980, 2006, and 2024. 4

Table S4: Proportion of immigrants (immigrants and Norwegian-born to immigrant parents) by origin, in %, in Norway, by region, during 1980–2024. 5

Figures 6

Figure S1: Age-standardised incidence rates of melanoma in Norway, by sex, during 1980–2024. 6

Figure S2: Age-specific incidence rates of melanoma in Norway, by sex and age group, during 1980–2024. 7

Tables

Table S1: Annual percent change (with 95% CI) in melanoma incidence in Norway, by age group, for males and females, during 1980-2024. Year intervals correspond to segments identified by joinpoint analysis.

Males			Females		
Age group	Years	APC (95% CI)	Age group	Years	APC (95% CI)
10-29 years	1980-1989	6·13 (-1·05, 56·67)	10-29 years	1980-1994	6·34 (3·63, 10·37)*
	1989-2024	-2·33 (-13·18, -1·33)*		1994-2003	-7·93 (-23·36, -2·87)*
30-39 years	1980-2024	-0·18 (-0·71, 0·35)		2003-2020	1·72 (-0·13, 17·67)
40-49 years	1980-1991	4·80 (2·82, 7·54)*		2020-2024	-16·57 (-36·97, -3·07)*
	1991-2002	-4·29 (-10·62, -2·25)*	30-39 years	1980-2024	0·02 (-0·28, 0·32)
	2002-2014	4·07 (2·22, 14·61)*	40-49 years	1980-1990	3·72 (1·72, 7·81)*
	2014-2024	-1·32 (-6·06, 0·82)		1990-1999	-3·46 (-12·77, -1·12)*
50-59 years	1980-1986	1·69 (-8·54, 7·03)		1999-2016	3·94 (2·91, 6·81)*
	1986-1989	17·73 (-2·13, 24·32)		2016-2024	-2·71 (-7·73, 0·08)
	1989-2004	-0·75 (-4·76, 5·13)	50-59 years	1980-1988	5·64 (1·77, 17·94)*
	2004-2024	3·21 (1·60, 4·93)*		1988-2004	-0·06 (-9·97, 1·60)
60-69 years	1980-1989	6·31 (3·47, 19·78)*		2004-2024	3·57 (2·31, 6·50)*
	1989-2024	2·82 (0·65, 3·20)*	60-69 years	1980-2024	3·03 (2·73, 3·33)*
70-79 years	1980-1995	6·91 (5·86, 8·71)*	70-79 years	1980-2024	3·87 (3·60, 4·14)*
	1995-2001	1·28 (-5·10, 4·82)	80+ years	1980-1999	2·59 (-2·06, 3·88)
	2001-2016	6·01 (4·91, 13·36)*		1999-2024	5·29 (4·44, 8·52)*
	2016-2024	1·25 (-3·45, 3·76)			
80+ years	1980-2024	5·89 (5·16, 6·59)*			
*p-value <0·05					

Table S2: Average annual percent change (with 95% CI) in melanoma incidence in Norway, by sex, age group and region, during 1980-2005 and 2006-2024.

Variable	Years	Males	Females
		AAPC (95% CI)	AAPC (95% CI)
**Age group**			
10-29	1980-2005	0·53 (-1·45, 2·65)	-0·05 (-1·57, 1·54)
	2006-2024	-0·85 (-5·07, 3·73)	-2·74 (-6·34, 1·17)
30-39	1980-2005	-0·90 (-1·95, 0·13)	-0·21 (-0·91, 0·48)
	2006-2024	-2·16 (-6·10, 3·97)	-0·80 (-2·02, 0·42)
40-49	1980-2005	0·43 (-0·45, 1·15)	1·31 (0·59, 2·17)*
	2006-2024	1·41 (-0·74, 3·82)	1·49 (0·04, 2·98)*
50-59	1980-2005	2·49 (0·86, 4·45)*	1·56 (0·33, 2·85)*
	2006-2024	3·18 (2·51, 3·86)*	3·32 (2·24, 4·40)*
60-69	1980-2005	3·72 (2·52, 4·91)*	2·99 (2·12, 3·85)*
	2006-2024	3·27 (1·91, 4·68)*	3·16 (2·21, 4·12)*
70-79	1980-2005	5·18 (4·33, 6·05)*	3·42 (2·62, 4·21)*
	2006-2024	3·85 (2·45, 5·40)*	4·71 (3·87, 5·56)*
80+	1980-2005	6·45 (4·31, 8·59)*	2·78 (1·92, 3·68)*
	2006-2024	5·33 (3·79, 7·06)*	5·11 (3·80, 6·38)*
**Region**			
South-East	1980-2005	1·80 (0·93, 2·84)*	1·34 (0·74, 2·01)*
	2006-2024	3·27 (2·74, 3·85)*	2·66 (1·14, 4·12)*
West	1980-2005	1·91 (1·14, 3·08)*	2·18 (1·28, 3·07)*
	2006-2024	2·86 (2·05, 3·67)*	2·38 (1·43, 3·34)*
Central	1980-2005	2·92 (2·05, 3·80)*	1·87 (0·69, 3·05)*
	2006-2024	3·85 (1·90, 5·87)*	3·35 (2·30, 4·43)*
North	1980-2005	4·64 (1·54, 7·80)*	1·17 (0·04, 2·32)*
	2006-2024	4·50 (3·09, 5·93)*	5·28 (3·50, 7·07)*

*p-value <0·05

Table S3: Region-specific incidence rates of melanoma per 100,000, by sex, in 1980, 2006, and 2024.

	**Males**			**Females**		
	1980	2006	2024	1980	2006	2024
**South-East**	14.2	23.1	37.8	16.8	25.6	34.2
**West**	14.5	26.2	38.9	15	29.9	37.3
**Central**	9.8	17.6	31.1	11.5	21.7	32
**North**	3.2	11.8	23.9	9.9	11	24.5

Table S4: Proportion of immigrants (immigrants and Norwegian-born to immigrant parents) by origin, in %, in Norway, by region, during 1980–2024.

		Immigrants and Norwegian-born to immigrant parents					
**Health region**	**Population group**	**1980**	**1990**	**2000**	**2010**	**2020**	**2024**
**South-East** (Agder, Telemark, Vestfold, Buskerud, Akershus, Oslo, Østfold, Innlandet)	**Total population**	**2,207,560**	**2,305,877**	**2,465,033**	**2,707,012**	**3,032,671**	**3,158,163**
	**Immigrants and Norwegian-born to immigrant parents**	**68,703 (3·1%)**	**121,197 (5·3%)**	**202,722 (8·2%)**	**380,853 (14·1%)**	**646,200 (21·3%)**	**758,493 (24·0%)**
	Europe	2·0%	2·5%	3·7%	6·1%	9·7%	11·3%
	Africa	0·1%	0·4%	0·8%	1·8%	3·0%	3·3%
	Asia	0·6%	1·9%	3·2%	5·5%	7·7%	8·4%
	North America	0·3%	0·3%	0·2%	0·2%	0·3%	0·3%
	Latin America and the Caribbean	0·1%	0·2%	0·3%	0·4%	0·6%	0·6%
	Oceania	0·0%	0·0%	0·0%	0·0%	0·1%	0·1%
**West** (Vestland, Rogaland)	**Total population**	**796,936**	**850,200**	**914,791**	**1,010,975**	**1,116,423**	**1,150,716**
	**Immigrants and Norwegian-born to immigrant parents**	**16,234 (2·0%)**	**28,945 (3·4%)**	**44,665 (4·9%)**	**98,312 (9·7%)**	**180,883 (16·2%)**	**211,335 (18·4%)**
	Europe	1·2%	1·7%	2·4%	5·2%	8·6%	10·0%
	Africa	0·0%	0·2%	0·4%	1·0%	2·0%	2·2%
	Asia	0·2%	0·9%	1·5%	2·9%	4·7%	5·1%
	North America	0·5%	0·4%	0·3%	0·2%	0·3%	0·3%
	Latin America and the Caribbean	0·1%	0·3%	0·3%	0·5%	0·6%	0·7%
	Oceania	0·0%	0·0%	0·0%	0·0%	0·1%	0·1%
**Central** (Trøndelag, Møre og Romsdal)	**Total population**	**605,908**	**616,765**	**634,345**	**674,591**	**733,940**	**753,580**
	**Immigrants and Norwegian-born to immigrant parents**	**5,884 (1·0%)**	**10,824 (1·8%)**	**20,088 (3·2%)**	**45,586 (6·8%)**	**94,959 (12·9%)**	**113,305 (15·0%)**
	Europe	0·7%	0·8%	1·7%	3·7%	6·8%	8·6%
	Africa	0·0%	0·1%	0·2%	0·7%	1·8%	1·9%
	Asia	0·1%	0·6%	1·0%	2·1%	3·7%	4·0%
	North America	0·1%	0·1%	0·1%	0·1%	0·2%	0·2%
	Latin America and the Caribbean	0·0%	0·1%	0·1%	0·2%	0·4%	0·4%
	Oceania	0·0%	0·0%	0·0%	0·0%	0·0%	0·0%
**North** (Finnmark, Troms, Nordland)	**Total population**	**468,496**	**460,274**	**464,328**	**465,621**	**484,546**	**487,744**
	**Immigrants and Norwegian-born to immigrant parents**	**4,107 (0·9%)**	**7,325 (1·6%)**	**14,986 (3·2%)**	**27,559 (5·9%)**	**57,219 (11·8%)**	**69,429 (14·2%)**
	Europe	0·7%	1·0%	2·1%	3·4%	6·3%	8·8%
	Africa	0·0%	0·1%	0·3%	0·8%	1·9%	1·8%
	Asia	0·0%	0·4%	0·7%	1·5%	3·3%	3·1%
	North America	0·1%	0·1%	0·1%	0·1%	0·1%	0·2%
	Latin America and the Caribbean	0·0%	0·0%	0·1%	0·1%	0·2%	0·3%
	Oceania	0·0%	0·0%	0·0%	0·0%	0·0%	0·0%
**Norway** (total)	**Total population**	**4,078,900**	**4,233,116**	**4,478,497**	**4,858,199**	**5,367,580**	**5,550,203**
	**Immigrants and Norwegian-born to immigrant parents**	**94,928 (2·3%)**	**168,291 (4·0%)**	**282,461 (6·3%)**	**552,310 (11·4%)**	**979,261 (18·2%)**	**1,152,562 (20·8%)**
	Europe	1·5%	1·9%	3·0%	5·3%	8·8%	10·4%
	Africa	0·1%	0·3%	0·6%	1·4%	2·6%	2·8%
	Asia	0·4%	1·3%	2·3%	4·1%	6·1%	6·7%
	North America	0·3%	0·2%	0·2%	0·2%	0·2%	0·3%
	Latin America and the Caribbean	0·1%	0·2%	0·2%	0·4%	0·5%	0·6%
	Oceania	0·0%	0·0%	0·0%	0·0%	0·0%	0·0%

Values are presented as N and percentages of the total population.

Figures

Figure S1: Age-standardised incidence rates of melanoma in Norway, by sex, during 1980–2024.

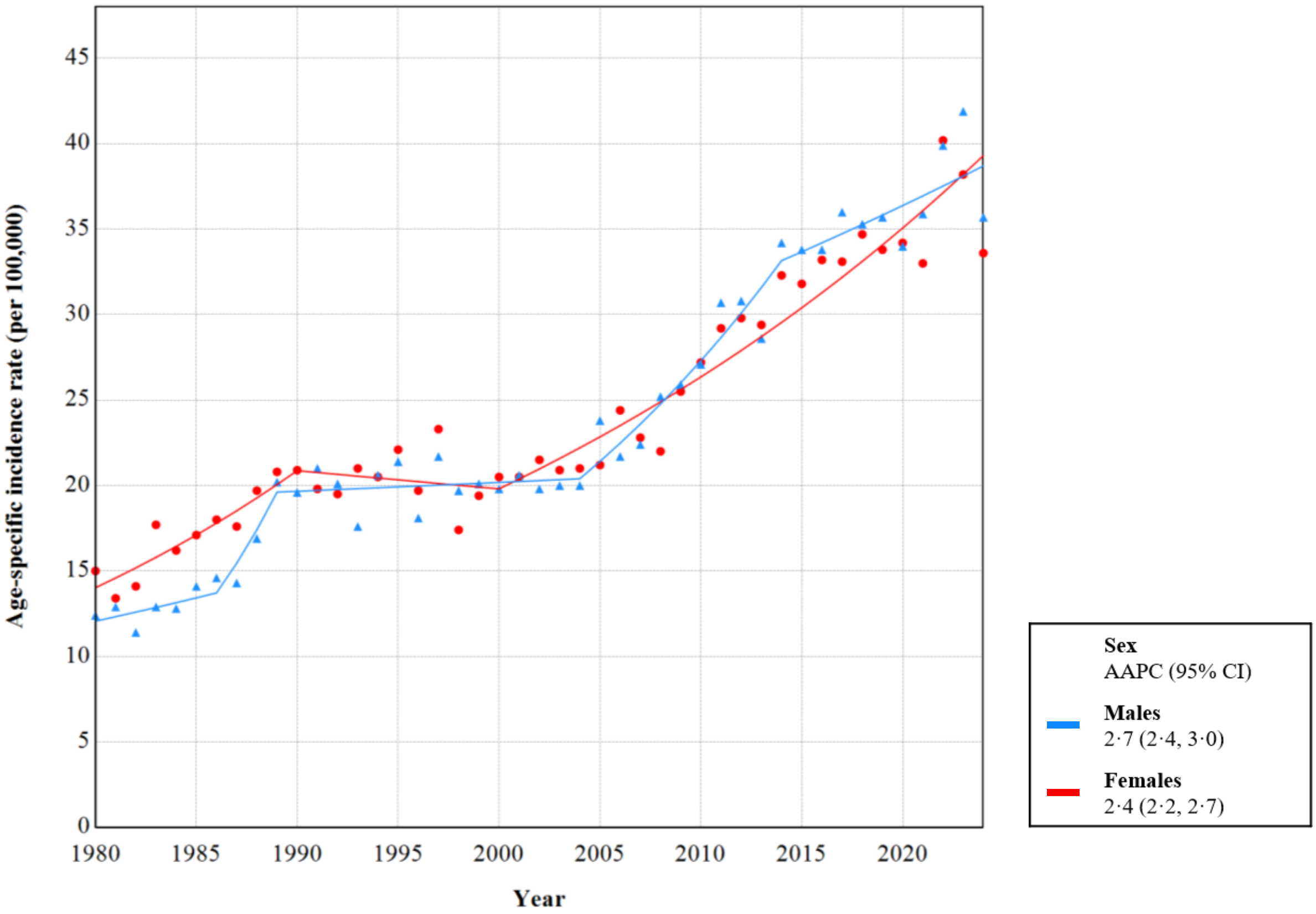

Figure S2: Age-specific incidence rates of melanoma in Norway, by sex and age group, during 1980–2024.

(A) Males

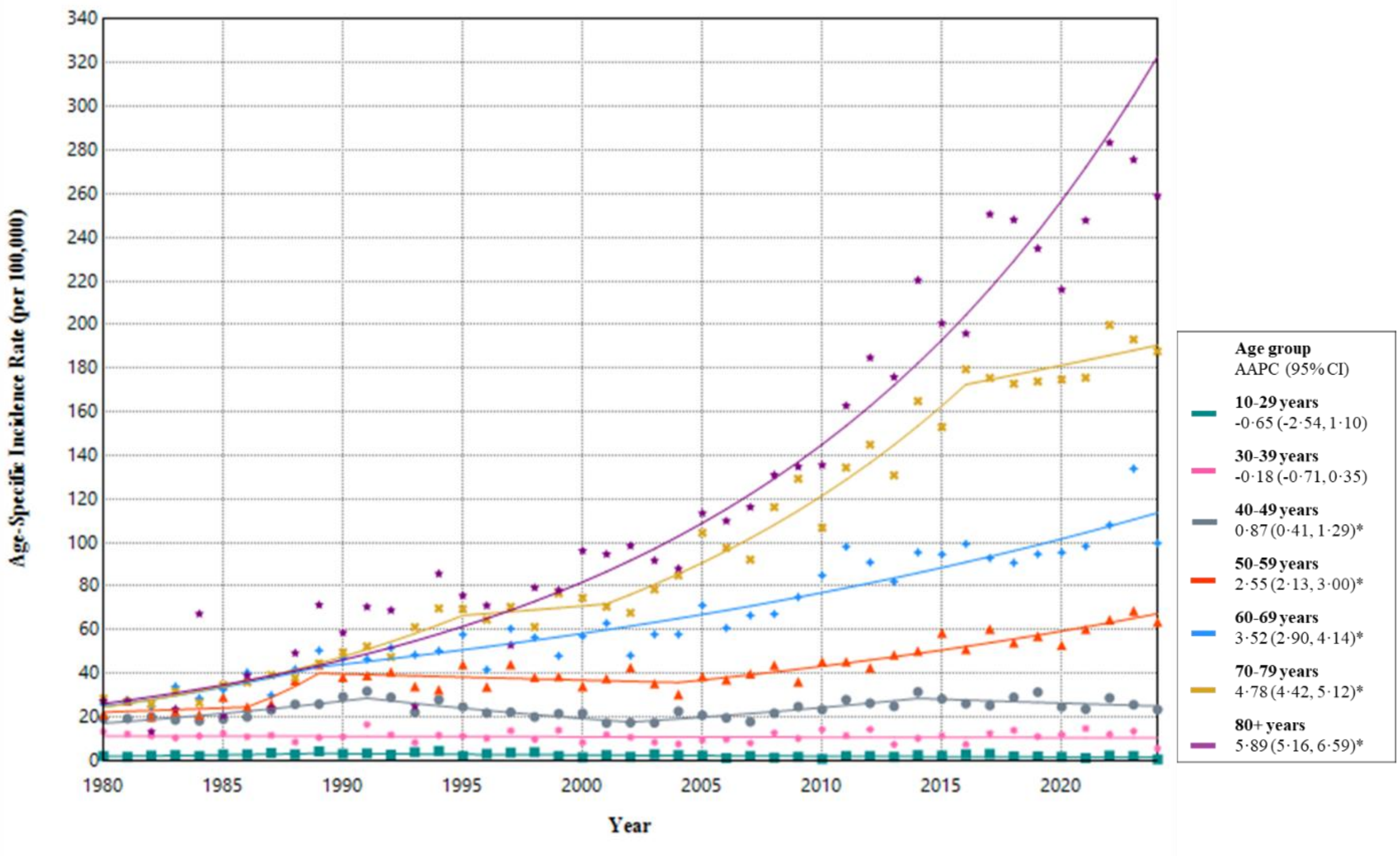

(B) Females

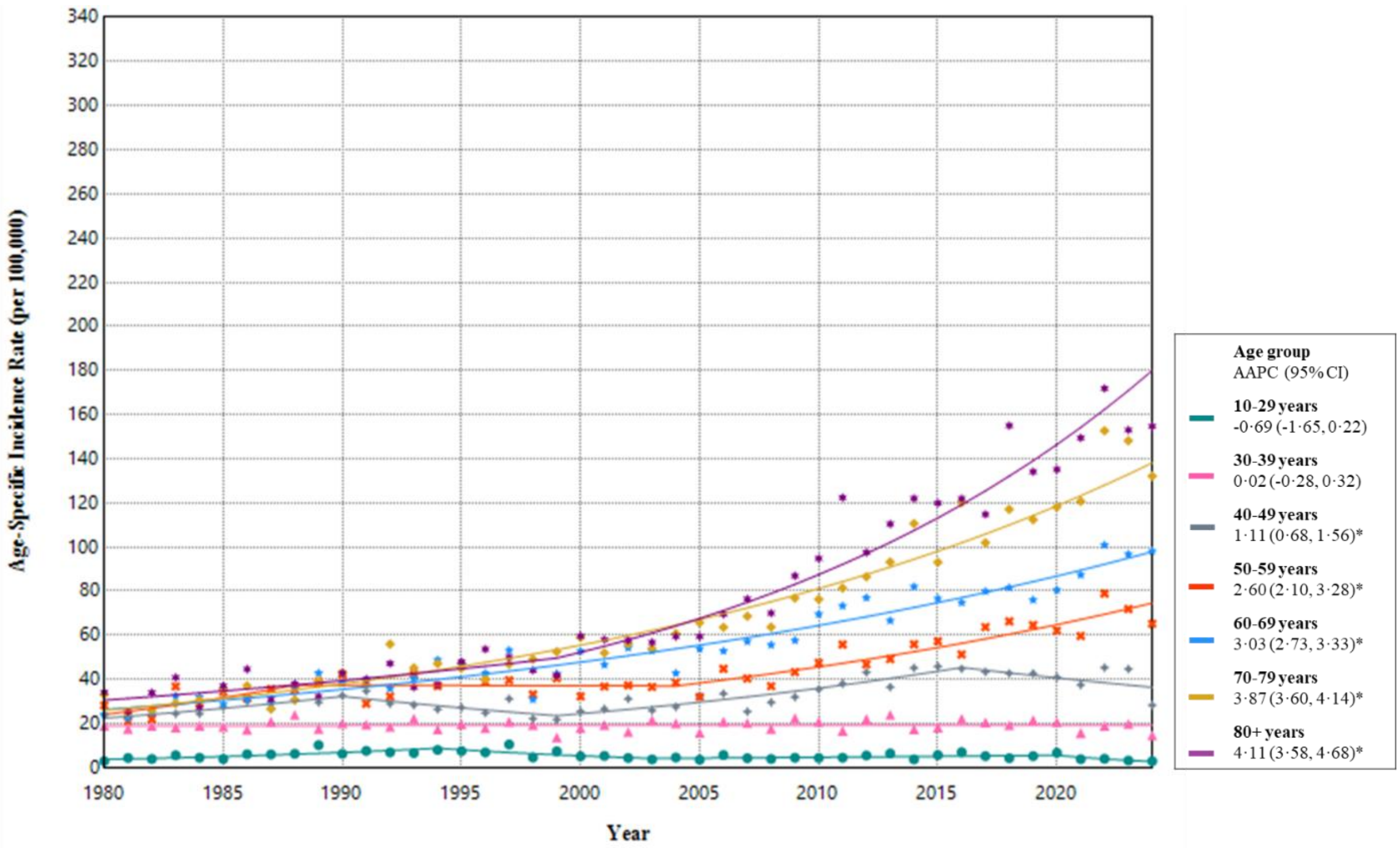

Supplement: Supplementary file 1 [file mmc1.pdf]
